# Sensor-Based Control for Collaborative Robots: Fundamentals, Challenges, and Opportunities

**DOI:** 10.3389/fnbot.2020.576846

**Published:** 2021-01-07

**Authors:** Andrea Cherubini, David Navarro-Alarcon

**Affiliations:** ^1^LIRMM, Univ Montpellier, CNRS, Montpellier, France; ^2^Department of Mechanical Engineering, The Hong Kong Polytechnic University, Hong Kong, Hong Kong

**Keywords:** robotics, human-robot collaboration (HRC), human-robot interaction (HRI), control systems (CS), visual servoing (VS)

## Abstract

The objective of this paper is to present a systematic review of existing sensor-based control methodologies for applications that involve direct interaction between humans and robots, in the form of either physical collaboration or safe coexistence. To this end, we first introduce the basic formulation of the sensor-servo problem, and then, present its most common approaches: vision-based, touch-based, audio-based, and distance-based control. Afterwards, we discuss and formalize the methods that integrate heterogeneous sensors at the control level. The surveyed body of literature is classified according to various factors such as: sensor type, sensor integration method, and application domain. Finally, we discuss open problems, potential applications, and future research directions.

## 1. Introduction

Robot control is a mature field: one that is already being heavily commercialized in industry. However, the methods required to regulate interaction and collaboration between humans and robots have not been fully established yet. These issues are the subject of research in the fields of physical human-robot interaction (pHRI) (Bicchi et al., 2008) and collaborative robotics (CoBots) (Colgate et al., 1996). The authors of De Luca and Flacco (2012) presented a paradigm that specifies three nested layers of consistent behaviors that the robot must follow to achieve safe pHRI:

- *Safety* is the first and most important feature in collaborative robots. Although there has been a recent push toward standardization of robot safety (e.g., the ISO 13482:2014 for robots and robotic devices; ISO 13482:2014, 2014), we are still in the initial stages. Safety is generally addressed through *collision avoidance* (with both humans or obstacles; Khatib, 1985), a feature that requires high reactivity (high bandwidth) and robustness at both the perception and control layers.
- *Coexistence* is the robot capability of sharing the workspace with humans. This includes applications involving a passive human (e.g., medical operations where the robot is intervening on the patients' body; Azizian et al., 2014), as well as scenarios where robot and human work together on the same task, without contact or coordination.
- *Collaboration* is the capability of performing robot tasks with direct human interaction and coordination. There are two modes for this: *physical* collaboration (with explicit and intentional contact between human and robot), and *contactless* collaboration (where the actions are guided by an exchange of information, e.g., in the form of body gestures, voice commands, or other modalities). Especially for the second mode, it is crucial to establish means for intuitive control by the human operators, which may be non-expert users. The robot should be proactive in realizing the requested tasks, and it should be capable of inferring the user's intentions, to interact more naturally from the human viewpoint.

All three layers are hampered by the unpredictability of human actions, which vary according to situations and individuals, complicating modeling (Phoha, 2014), and use of classic control.

In the robotics literature, two major approaches for task execution have emerged: *path/motion planning* (La Valle, 2006) and *sensor-based control* (Chaumette and Hutchinson, 2006). The *planning* methods rely on a priori knowledge of the future robot and environment states over a time window. Although they have proved their efficiency in well-structured applications, these methods are hardly applicable to human-robot collaboration, because of the unpredictable and dynamic nature of humans. It is in the authors' view that *sensor-based control* is more efficient and flexible for pHRI, since it closes the perception-to-action loop at a lower level than path/motion planning. Note also that sensor-based control strategies strongly resemble the processes of our central nervous system (Berthoz, 2002), and can trace their origins back to the servomechanism problem (Davison and Goldenberg, 1975). The most known example is image-based visual servoing (Chaumette and Hutchinson, 2006) which relies directly on visual feedback to control robot motion, without requiring a cognitive layer nor a precise model of the environment.

The aim of this article is to survey the current state of art in *sensor-based control, as a means to facilitate the interaction between robots, humans, and surrounding environments*. Although we acknowledge the need for other techniques within a complete human-robot collaboration framework (e.g., path planning as mentioned, machine learning, etc.), here we review and classify the works which exploit sensory feedback to directly command the robot motion.

The timing and relevance of this survey is twofold. On one hand, while there have been previous reviews on topics such as (general) human-robot collaboration (Ajoudani et al., 2017; Villani et al., 2018) and human-robot safety (Haddadin et al., 2017), there is no specific review on the use of *sensor-based control* for human-robot collaborative tasks. On the other hand, we introduce a *unifying* paradigm for designing controllers with four sensing modalities. This feature gives our survey a valuable tutorial-like nature.

The rest of this manuscript is organized as follows: Section 2 presents the basic formulation of the sensor-based control problem; Section 3 describes the common approaches that integrate multiple sensors at the control level. Section 4 provides several classifications of the reviewed works. Section 5 presents insights and discusses open problems and areas of opportunity. Conclusions are given in section 6.

## 2. Sensing Modalities for Control

**Figure 1:**
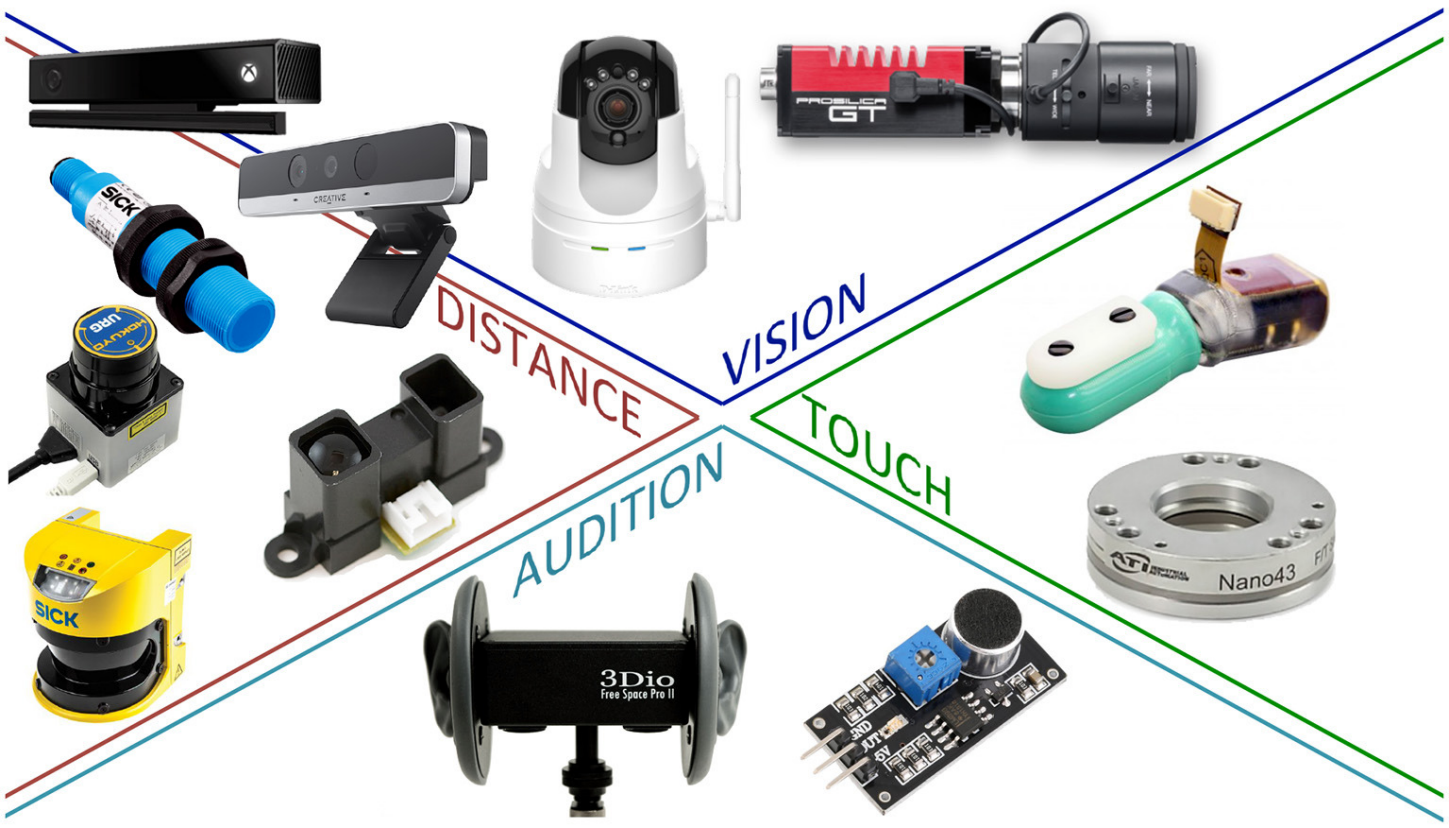
Examples of artificial sensors. Clockwise from the top left: Microsoft Kinect® and Intel Realsense® (vision and distance), Sony D-Link DCS-5222L® and AVT GT® (vision), Syntouch BioTac® and ATI Nano 43® (touch), sound sensor LM393® and 3Dio Free Space Pro II® Binaural Microphone (audition), proximity sensor Sharp GP2Y0A02YK0F®, Laser SICK®, Hokuyo URG®, and proximity sensor SICK CM18-08BPP-KC1® (distance). Note that Intel Realsense® and Microsoft Kinect® provide both the senses of vision and of distance.

Recent developments on bio-inspired measurement technologies have made sensors affordable and lightweight, easing their use on robots. These sensors include RGB-D cameras, tactile skins, force/moment transducers, etcetera (see Figure 1). The works reviewed here rely on different combinations of sensing modalities, depending on the task at stake. We consider the following four robot senses:

- *Vision*. This includes methods for processing and understanding images, to produce numeric or symbolic information reproducing human sight. Although image processing is complex and computationally expensive, the richness of this sense is unique. Robotic vision is fundamental for understanding the environment and human intention, so as to react accordingly.
- *Touch*. In this review, touch includes both *proprioceptive force* and *tact*, with the latter involving *direct* physical contact with an external object. *Proprioceptive force* is analogous to the sense of muscle force (Proske and Gandevia, 2012). The robot can measure it either from the joint position errors or via torque sensors embedded in the joints; it can then use both methods to infer and adapt to human intentions, by relying on force control (Raibert and Craig, 1981; Hogan, 1985; Morel et al., 1998; Villani and De Schutter, 2008). Human *tact* (somatosensation), on the other hand, results from activation of neural receptors, mostly in the skin. These have inspired the design of artificial tactile skins (Wettels et al., 2008; Schmitz et al., 2011), thoroughly used for human-robot collaboration.
- *Audition*. In humans, localization of sound is performed by using binaural audition (i.e., two ears). By exploiting auditory cues in the form of level/time/phase differences between left and right ears we can determine the source's horizontal position and its elevation (Rayleigh, 1907). Microphones artificially emulate this sense, and allow robots to “blindly” locate sound sources. Although robotic hearing typically uses two microphones mounted on a motorized head, other non-biological configurations exist, e.g., a head instrumented with a single microphone or an array of several omni-directional microphones (Nakadai et al., 2006).
- *Distance*. This is the only sense among the four that humans cannot directly measure. Yet, numerous examples exist in the mammal kingdom (e.g., bats and whales), in the form of echolocation. Robots measure distance with *optical* (e.g., infrared or lidar), *ultrasonic*, or *capacitive* (Göger et al., 2010) sensors. The relevance of this particular “sense” in human-robot collaboration is motivated by the direct relationship existing between the distance from obstacles (here, the human) and safety.

Roboticists have designed other bio-inspired sensors, to *smell* (see Kowadlo and Russell, 2008 for a comprehensive survey and Russell, 2006; Gao et al., 2016; Rahbar et al., 2017 for 3D tracking applications) and *taste* (Shimazu et al., 2007; Kobayashi et al., 2010; Ha et al., 2015). However, in our opinion, artificial *smell* and *taste* are not yet mature enough for human-robot collaboration. Most of the current work on these senses is for localization/identification of hazardous gases/substances. It is also worth mentioning the increasing popularity of physiological signals for controlling robots. These include, for example, Electromyography and Brain-Computer Interfaces (Ajoudani et al., 2017). Albeit promising, these technologies generally provide unilateral (from human to robot) control, without feedback loop closure. For these reasons, this review will focus only on the four senses mentioned above, namely vision, touch, audition, and distance.

## 3. Sensor-Based Control

### 3.1. Formulation of Sensor-Based Control

Sensor-based control aims at deriving the robot control input **u** (operational space velocity, joint velocity, displacement, etc.) that minimizes a *trajectory* error **e** = **e**(**u**), which can be estimated by sensors and depends on **u**. A general way of formulating this controller [accounting for actuation redundancy dim(**u**) > dim(**e**), sensing redundancy dim(**u**) < dim(**e**), and task constraints] is as the quadratic minimization problem:

(1) $$\begin{array}{r} \begin{array}{c} \text{u}=\underset{\text{u}}{arg min}\;\frac{1}{2}||\text{e}\left(\text{u}\right)|{|}^{2}\\ \text{subject to task constraints.} \end{array} \end{array}$$

This formulation encompasses the classic *inverse kinematics* problem (Whitney, 1969) of controlling the robot joint velocities ($\text{u}=\overset{∙}{\text{q}}$), so that the end-effector operational space position **x** converges to a desired value x^*^. By defining the desired end-effector rate as ${\overset{∙}{\text{x}}}^{*}=-\lambda \left(\text{x}-{\text{x}}^{*}\right)$, for λ > 0, and setting $\text{e}=\text{J}\overset{∙}{\text{q}}-{\overset{∙}{\text{x}}}^{*}$ for **J** = ∂**x**/∂**q** as the Jacobian matrix, it is easy to show that the solution to (1) (in the absence of constraints) is $\overset{∙}{\text{q}}={\text{J}}^{+}{\overset{∙}{\text{x}}}^{*}$, with **J**^+^ the generalized inverse of **J**. This leads to the set-point controller^1^:

(2) $$\begin{array}{r} \overset{∙}{\text{q}}=-{\text{J}}^{+}\lambda \left(\text{x}-{\text{x}}^{*}\right). \end{array}$$

**Figure 2:**
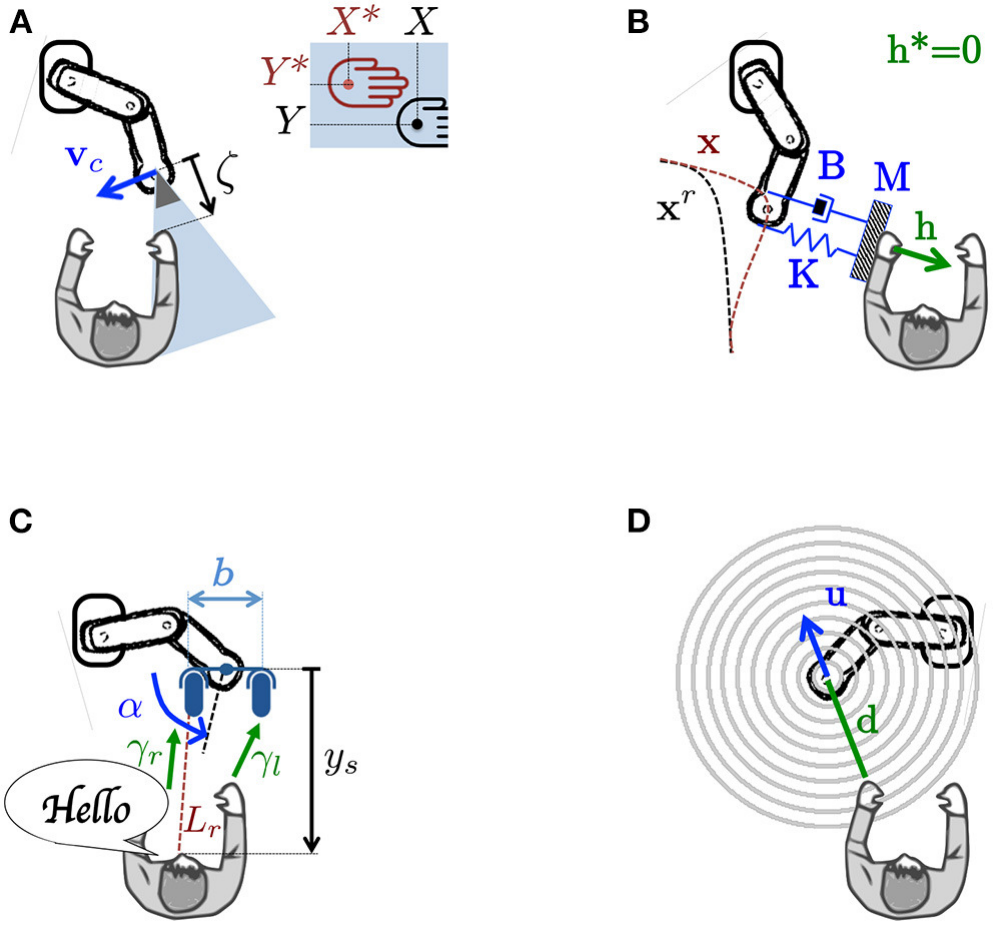
Examples of four sensor-based servo controllers. **(A)** Visual servoing: the user hand is centered in the camera image. **(B)** Indirect force control: by applying a wrench, the user deviates the contact point away from a reference trajectory. **(C)** Audio-based control: a microphone rig is automatically oriented toward the sound source (the user's mouth). **(D)** Distance-based control: the user acts as a repulsive force, related to his/her distance from the robot.

In the following sections, we show how each of the four senses (*vision, touch, audition* and *distance*) has been used for robot control, either with (1), or with similar techniques. Figure 2 shows relevant variables for the four cases. For simplicity, we assume there are no constraints in (1), although off-the-shelf quadratic programming solvers (Nocedal and Wright, 2000) could account for them.

### 3.2. Visual Servoing

#### 3.2.1. Formulation

Visual servoing refers to the use of vision to control the robot motion (Chaumette and Hutchinson, 2006). The camera may be mounted on a moving part of the robot, or fixed in the workspace. These two configurations are referred to as “eye-in-hand” and “eye-to-hand” visual servoing, respectively. The error **e** is defined with regards to some image features, here denoted by **s**, to be regulated to a desired configuration s^*^ (**s** is analogous to **x** in the inverse kinematic formulation above). The visual error is:

(3) $$\begin{array}{r} \text{e}=\overset{∙}{\text{s}}-{\overset{∙}{\text{s}}}^{*}. \end{array}$$

Visual servoing schemes are called *image-based* if **s** is defined in image space, and *position-based* if **s** is defined in the 3D operational space. Here we only briefly recall the image-based approach (on its eye-in-hand modality), since the position-based one consists in projecting the task from the image to the operational space to obtain **x** and then apply (2).

The simplest image-based controller uses **s** = [*X, Y*]^⊤^, with *X* and *Y* as the coordinates of an image pixel, to generate **u** that drives **s** to a reference s^*^ = [X^*^, Y^*^]⊤ (in Figure 2A the centroid of the human hand). This is done by defining **e** as:

(4) $$\begin{array}{r} \overset{∙}{\text{s}}-{\overset{∙}{\text{s}}}^{*}=\left[\begin{array}{c} Ẋ-{Ẋ}^{*}\\ Ẏ-{Ẏ}^{*} \end{array}\right],\text{with}{\overset{∙}{\text{s}}}^{*}=-\lambda \left[\begin{array}{c} X-X^{*}\\ Y-Y^{*} \end{array}\right] \end{array}$$

If we use the camera's 6D velocity as the control input **u** = **v**_*c*_, the image Jacobian matrix^2^ relating [*Ẋ*, *Ẏ*]^⊤^ and **u** is:

(5) $$\begin{array}{r} {\text{J}}_{v}=\left[\begin{array}{cccccc} -\frac{1}{\zeta } & 0 & \frac{X}{\zeta } & XY & -1-X^{2} & Y\\ 0 & -\frac{1}{\zeta } & \frac{Y}{\zeta } & 1+Y^{2} & -XY & -X \end{array}\right], \end{array}$$

where ζ denotes the depth of the point with respect to the camera. In the absence of constraints, the solution of (1) is:

(6) $$\begin{array}{r} {\text{v}}_{c}=-{\text{J}}_{v}^{+}\lambda \left[\begin{array}{c} X-X^{*}\\ Y-Y^{*} \end{array}\right]. \end{array}$$

#### 3.2.2. Application to Human-Robot Collaboration

Humans generally use vision to teach the robot relevant configurations for collaborative tasks. For example, Cai et al. (2016) demonstrate an application where a human operator used a QR code to specify the target poses for a 6 degrees-of-freedom (dof) robot arm. In Gridseth et al. (2016), the user provided target tasks via a tablet-like interface that sent the robot the desired reference view; here, the human specified various motions such as point-to-point, line-to-line, etc., that were automatically performed via visual feedback. The authors of Gridseth et al. (2015) presented a grasping system for a tele-operated dual arm robot, where the user specified the object to be manipulated, and the robot completed the task using visual servoing.

Assistive robotics represents another very common application domain for visual servoing. The motion of robotic wheelchairs has been semi-automated at various degrees. For instance, Narayanan et al. (2016) presented a corridor following method that exploited the projection of parallel lines. In this work, the user provided target directions with a haptic interface, and the robot corrected the trajectories with visual feedback. Other works have focused on mobile manipulation. The authors of Tsui et al. (2011) developed a vision-based controller for a robotic arm mounted on a wheelchair; in this work, the user manually specified the object to be grasped and retrieved by the robot. A similar approach was reported in Dune et al. (2008), where the desired poses were provided with “clicks” on an screen interface.

Medical robotics is another area that involves sensor-based interactions between humans and robots, and where vision has huge potential (see Azizian et al., 2014 for a comprehensive review). For example, the authors of Agustinos et al. (2014) developed a laparoscopic camera, which regulated its pan/tilt motions to track human-held instruments.

### 3.3. Touch (or Force) Control

#### 3.3.1. Formulation

Touch (or force) control requires the measurement of one or multiple (in the case of tactile skins) *wrenches* **h**, which are (at most) composed of three translational forces, and three torques; **h** is fed to the controller that moves the robot so that it exerts a desired interaction force with the human or environment. Force control strategies can be grouped into the following two classes (Villani and De Schutter, 2008):

- *Direct* control regulates the contact wrench to obtain a desired wrench h^*^. Specifying h^*^ requires an explicit model of the task and environment. A widely adopted strategy is hybrid position/force control (Raibert and Craig, 1981), which regulates the velocity and wrench along unconstrained and constrained task directions, respectively. Referring to (1), this is equivalent to setting
  (7) $$\begin{array}{r} \text{e}=\text{S}\left(\overset{∙}{\text{x}}-{\overset{∙}{\text{x}}}^{*}\right)+\left(\text{I}-\text{S}\right)\left(\text{h}-{\text{h}}^{*}\right), \end{array}$$
  with **S** = **S**^⊤^≥0 a binary diagonal selection matrix, and **I** as the identity matrix. Applying a motion **u** that nullifies **e** in (7) guarantees that the components of $\overset{∙}{\text{x}}$ (respectively **h**) specified via **S** (respectively **I**−**S**) converge to ${\overset{∙}{\text{x}}}^{*}$ (respectively h^*^).
- *Indirect* control (illustrated in Figure 2B) does not require an explicit force feedback loop. To this category belong *impedance control* and its dual *admittance control* (Hogan, 1985). It consists in modeling the deviation of the contact point from a reference trajectory **x**^*r*^(*t*) associated to the desired h^*^, via a virtual mechanical impedance with adjustable parameters (inertia **M**, damping **B**, and stiffness **K**). Referring to (1), this is equivalent to setting:
  (8) $$\begin{array}{r} \text{e}=\text{M}\left(\ddot{\text{x}}-{\ddot{\text{x}}}^{r}\right)+\text{B}\left(\overset{∙}{\text{x}}-{\overset{∙}{\text{x}}}^{r}\right)+\text{K}\left(\text{x}-{\text{x}}^{r}\right)-\left(\text{h}-{\text{h}}^{*}\right). \end{array}$$
  Here, **x** represents the “deviated” contact point pose, with $\overset{∙}{\text{x}}$ and $\ddot{\text{x}}$ as time derivatives. When **e** = **0**, the displacement **x**−**x**^*r*^ responds as a mass-spring-damping system under the action of an external force **h**−h^*^. In most cases, **x**^*r*^(*t*) is defined for motion in free space (h^*^ = **0**). The general formulation in (1) and (8) can account for both impedance control (**x** is measured and **u** = **h**) and admittance control (**h** measured and **u** = **x**).

#### 3.3.2. Application to Human-Robot Collaboration

The authors of Bauzano et al. (2016) used direct force control for collaborative human-robot laparoscopic surgery. In their method, the instruments are controlled with a hybrid position/force approach. In Cortesao and Dominici (2017), a robot regulated the applied forces onto a beating human heart. Since the end-effector's 3 linear dof were fully-constrained, position control could not be performed, i.e., **S** = **0** in (7).

A drawback of direct control is that it can realize only the tasks which can be described via constraint surfaces. If their location is unknown and/or the contact geometry is complex—as often in human-robot collaboration—indirect control is more suited since: (i) it allows to define a priori how the robot should react to unknown external force disturbances, (ii) it can use a reference trajectory **x**^*r*^(*t*) output by another sensor (e.g., vision). In the next paragraph, we review indirect force control methods.

By sensing force, the robot can infer the motion commands (e.g., pushing, pulling) from the human user. For example, Maeda et al. (2001) used force sensing and human motion estimation (based on minimum jerk) within an indirect (admittance) control framework for cooperative manipulation. In Suphi Erden and Tomiyama (2010) and Suphi Erden and Maric (2011), an assistant robot suppressed involuntary vibrations of a human, who controlled direction and speed of a welding operation. By exploiting kinematic redundancy, Ficuciello et al. (2013) also addressed a manually guided robot operation. The papers (Bussy et al., 2012; Wang et al., 2015) presented admittance controllers for two-arm robots moving a table in collaboration with a human. In Baumeyer et al. (2015), a human controlled a medical robot arm with an admittance controller. Robot *tele-operation* is another common human-robot collaboration application where force feedback plays a crucial role; see Passenberg et al. (2010) for a comprehensive review on the topic.

All these works relied on local force/moment measurements. Up to this date, tactile sensors and skins (measuring the wrench along the robot body, see Argall and Billard, 2010 for a review) have been used for object exploration (Natale and Torres-Jara, 2006) or recognition (Abderrahmane et al., 2018), but not for control as expressed in (1). One reason is that they are at a preliminary design stage, which still requires complex calibration (Del Prete et al., 2011; Lin et al., 2013) that constitutes a research topic per se. An exception is Li et al. (2013), which presented a method that used tactile measures within (1). Similarly, in Zhang and Chen (2000), tactile sensing was used to regulate interaction with the environment. Yet, neither of these works considered pHRI. In our opinion, there is huge potential in the use of skins and tactile displays for human-robot collaboration.

### 3.4. Audio-Based Control

#### 3.4.1. Formulation

The purpose of audio-based control is to locate the sound source, and move the robot toward it. For simplicity, we present the two-dimensional binaural (i.e., with two microphones) configuration in Figure 2C, with the angular velocity of the microphone rig as control input: $\text{u}=\overset{∙}{\alpha }$. We hereby review the two most popular methods for defining error **e** in (1): Interaural Time Difference (ITD) and Interaural Level Difference (ILD)^3^. The following is based on Magassouba et al. (2016b):

- *ITD-based aural servoing* uses the difference τ between the arrival times of the sound on each microphone; τ must be regulated to a desired τ^*^. The controller can be represented with (1), by setting $\text{e}=\overset{∙}{\tau }-{\overset{∙}{\tau }}^{*}$, with the desired rate ${\overset{∙}{\tau }}^{*}=-\lambda \left(\tau -\tau^{*}\right)$ (to obtain set-point regulation to τ^*^). Feature τ can be derived in real-time by using standard cross-correlation of the signals (Youssef et al., 2012). Under a far field assumption:
  (9) $$\begin{array}{r} \text{e}=\overset{∙}{\tau }-{\overset{∙}{\tau }}^{*}=-\left(\sqrt{{\left(b/c\right)}^{2}-\tau^{2}}\right)\text{u}-{\overset{∙}{\tau }}^{*} \end{array}$$
  with *c* the sound celerity and *b* the microphones baseline. From (9), the *scalar* ITD Jacobian is: ${\text{J}}_{\tau }=-\sqrt{{\left(b/c\right)}^{2}-\tau^{2}}$. The motion that minimizes **e** is:
  (10) $$\begin{array}{r} \text{u}=-\lambda {\text{J}}_{\tau }^{-1}\left(\tau -\tau^{*}\right), \end{array}$$
  which is locally defined for α ∈ (0, π), to ensure that |**J**_τ_|≠0.
- *ILD-based aural servoing* uses ρ, the difference in intensity between the left and right signals. This can be obtained in a time window of size *N* as ρ = *E*_*l*_/*E*_*r*_, where the $E_{l,r}=\overset{N}{\underset{n=0}{\sum }}\gamma_{l,r}{\left[n\right]}^{2}$ denote the signals' sound energies and the γ_*l,r*_[*n*] are the intensities at iteration *n*. To regulate ρ to a desired ρ^*^, one can set $\text{e}=\overset{∙}{\rho }-{\overset{∙}{\rho }}^{*}$ with ${\overset{∙}{\rho }}^{*}=-\lambda \left(\rho -\rho^{*}\right)$. Assuming spherical propagation and slowly varying signal:
  (11) $$\begin{array}{r} \text{e}=\overset{∙}{\rho }-{\overset{∙}{\rho }}^{*}=\frac{y_{s}\left(\rho +1\right)b}{L_{r}^{2}}\text{u}-{\overset{∙}{\rho }}^{*} \end{array}$$
  where *y*_*s*_ is the sound source frontal coordinate in the moving auditory frame, and *L*_*r*_ the distance between the right microphone and the source. From (11), the *scalar* ILD Jacobian is ${\text{J}}_{\rho }=y_{s}\left(\rho +1\right)b/L_{r}^{2}$. The motion that minimizes **e** is:
  (12) $$\begin{array}{r} \text{u}=-\lambda {\text{J}}_{\rho }^{-1}\left(\rho -\rho^{*}\right) \end{array}$$
  where ${\text{J}}_{\rho }^{-1}$ is defined for sources located in front of the rig. In contrast with ITD-servoing, here the source location (i.e., *y*_*s*_ and *L*_*r*_) must be known or estimated.

While the methods above only control the angular velocity of the rig ($\text{u}=\overset{∙}{\alpha }$), Magassouba extended both to also regulate 2D translations of a mobile platform (ITD in Magassouba et al., 2015, 2016c and ILD in Magassouba et al., 2016a).

#### 3.4.2. Application to Human-Robot Collaboration

Due to the nature of this sense, audio-based controllers are mostly used in contact-less applications, to enrich other senses (e.g., force, distance) with sound, or to design intuitive human-robot interfaces.

Audio-based control is currently (in our opinion) an underdeveloped research area with great potential for human-robot collaboration, e.g., for tracking a speaker. Besides the cited works (Magassouba et al., 2015, 2016a,b,c), that closely followed the framework of section 3, others have formulated the problem differently. For example, the authors of Kumon et al. (2003, 2005) proposed a linear model to describe the relation between the pan motion of a robot head and the difference of intensity between its two microphones. The resulting controllers were much simpler than (10) and (12). Yet, their operating range was smaller, making them less robust than their—more analytical—counterparts.

### 3.5. Distance-Based Control

#### 3.5.1. Formulation

The simplest (and most popular) distance-based controller is the artificial potential fields method (Khatib, 1985). Despite being prone to local minima, it has been thoroughly deployed both on manipulators and on autonomous vehicles for obstacle avoidance. it is acceptable that a collaborative robot stops (e.g., because of local minima) as long as it avoids the human user. The potential fields method consists in modeling each obstacle as a source of repulsive forces, related to the robot distance from the obstacle (see Figure 2D). All the forces are summed up resulting in a velocity in the most promising direction. Given **d**, the position of the nearest obstacle in the robot frame, the original version (Khatib, 1985) consists in applying operational space velocity

(13) $$u=\{\begin{array}{ll} \lambda \left(\frac{1}{\left\|d\right\|}-\frac{1}{d_{o}}\right)\frac{d}{\|d{\|}^{2}} & \text{ if }\|d\|<d_{o},\\ 0 & \text{ otherwise.} \end{array}$$

Here *d*_*o*_ > 0 is the (arbitrarily tuned) minimal distance required for activating the controller. Since the quadratic denominator in (13) yields abrupt accelerations, more recent versions adopt a linear behavior. Referring to (1), this can be obtained by setting $\text{e}=\overset{∙}{\text{x}}-{\overset{∙}{\text{x}}}^{*}$ with ${\overset{∙}{\text{x}}}^{*}=\lambda \left(1-d_{0}/||\text{d}||\right)\text{d}$ as reference velocity:

(14) $$\begin{array}{r} \text{e}=\overset{∙}{\text{x}}-\lambda \left(1-\frac{d_{0}}{\left||\text{d}|\right|}\right)\text{d}. \end{array}$$

By defining as control input $\text{u}=\overset{∙}{\text{x}}$, the solution to (1) is:

(15) $$\begin{array}{r} \text{u}=\lambda \left(1-\frac{d_{0}}{\left||\text{d}|\right|}\right)\text{d}. \end{array}$$

#### 3.5.2. Application to Human-Robot Collaboration

Many works have used this (or similar) distance-based methods for pHRI. To avoid human-robot collisions, the authors of De Santis et al. (2007) applied the controller (15) by estimating the distance **d** between a human head and a robot with vision. Recently, these approaches have been boosted by the advent of 3D vision sensors (e.g., the Microsoft Kinect and Intel RealSense), which enable both vision and distance control. The authors of Flacco et al. (2012) designed a Kinect-based distance controller (again, for human collision avoidance) with an expression similar to (15), but smoothed by a sigmoid.

*Proximity servoing* is a similar technique, which regulates—via capacitive sensors—the distance between the robot surface and the human. In Schlegl et al. (2013), these sensors modified the position and velocity of a robot arm when a human approached it, to avoid collisions. The authors of Bergner et al. (2017), Leboutet et al. (2016), and Dean-Leon et al. (2017) developed a new capacitive skin for a dual-arm robot. They designed a collision avoidance method based on an admittance model similar to (8), which relied on the joint torques (measured by the skin) to control the robot motion.

## 4. Integration of Multiple Sensors

In section 3, we presented the most common sensor-based methods used for collaborative robots. Just like natural senses, artificial senses provide complementary information about the environment. Hence, to effectively perform a task, the robot should measure (and use for control) multiple feedback modalities. In this section, we review various methods for integrating multiple sensors in a unique controller.

Inspired by how humans merge their percepts (Ernst and Banks, 2002), researchers have traditionally fused heterogeneous sensors to estimate the state of the environment. This can be done in the sensors' Cartesian frames (Smits et al., 2008) by relying on an Extended Kalman Filter (EKF) (Taylor and Kleeman, 2006). Yet the sensors must be related to a single quantity, which is seldom the case when measuring different physical phenomena (Nelson and Khosla, 1996). An alternative is to use the sensed feedback directly in (1). This idea, proposed for position-force control in Raibert and Craig (1981) and extended to vision in Nelson et al. (1995), brings new challenges to the control design, e.g., sensor synchronization, task compatibility, and task representation. For instance, the designer should take care when transforming 6 D velocities or wrenches to a unique frame. This requires (when mapping from frame *A* to frame *B*) multiplication by

(16) BVA=[RBA[tBA]×BRA03RBA]

for a velocity, and by ${}^{B}{\text{V}}_{A}^{⊤}$ for a wrench. In (16), ${}^{B}{\text{R}}_{A}$ is the rotation matrix from *A* to *B* and [tBA]× the skew-symmetric matrix associated to translation ${}^{B}{\text{t}}_{A}$.

**Figure 3:**
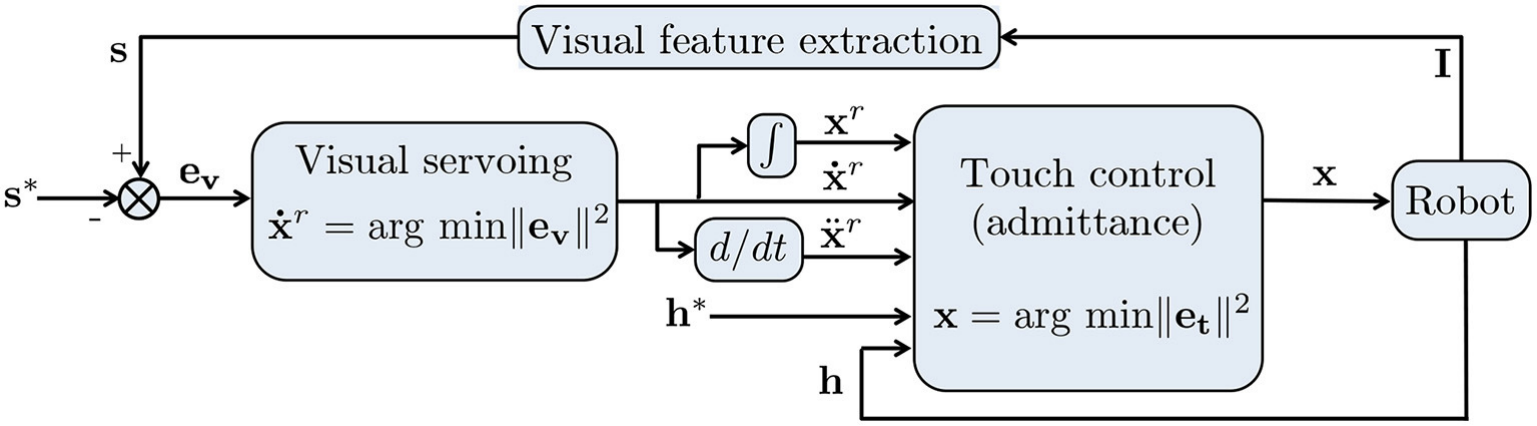
The most common scheme for *shared* vision/touch (admittance) control, used in Morel et al. (1998), Agravante et al. (2013, 2014). The goal is to obtain desired visual features s^*^ and wrench h^*^, based on current image **I** and wrench **h**. The *outer* visual servoing loop based on error (3) outputs a reference velocity ${\overset{∙}{\text{x}}}^{r}$ that is then deformed by the *inner* admittance control loop based on error (8), to obtain the desired robot position **x**.

According to Nelson et al. (1995), the three methods for combining *N* sensors within a controller are:

- *Traded*: the sensors control the robot one at a time. Predefined conditions on the task trigger the switches:
  (17) $$u=\left\{ \begin{array}{l} \underset{u}{\arg\;\min}\;\|e_{1}(u){\|}^{2}\text{if }(condition\;1)=\text{true,}\\ \vdots \\ \underset{u}{\arg\;\min}\;\|e_{N}(u){\|}^{2}\text{if }(condition\;N)=\text{true.} \end{array}\right.$$
- *Shared*: All sensors control the robot throughout operation. A common way is via nested control loops, as shown—for shared vision/touch control—in Figure 3. Researchers have used at most two loops, denoted *o* for outer and *i* for inner loop:
  (18) $$\begin{array}{r} \text{u}=\underset{\text{u}}{arg min}\;||{\text{e}}_{i}\left(\text{u},{\text{u}}_{o}\right)|{|}^{2}\\ \;\text{such that}{\;\text{u}}_{o}=\underset{{\text{u}}_{o}}{arg min}\;||{\text{e}}_{o}\left({\text{u}}_{o}\right)|{|}^{2}. \end{array}$$
  In the example of Figure 3: **u** = **x**, ${\text{u}}_{o}={\overset{∙}{\text{x}}}^{r}$, **e**_*o*_ = **e**_**v**_ applying (3) and **e**_*i*_ = **e**_**t**_ applying (8).
- *Hybrid*: the sensors act simultaneously, but on different axes of a predefined Cartesian *task-frame* (Baeten et al., 2003). The directions are selected by binary diagonal matrices **S**_*j*_, *j* = 1, …, *N* with the dimension of the task space, and such that $\overset{N}{\underset{j=1}{\sum }}\text{S}=\text{I}$:
  (19) $$\begin{array}{r} \text{u}=\underset{\text{u}}{arg min}\;||\overset{N}{\underset{j=1}{\sum }}{\text{S}}_{j}{\text{e}}_{j}\left(\text{u}\right)|{|}^{2}. \end{array}$$
  To express all **e**_*j*_ in the same task frame, one should apply ${}^{B}{\text{V}}_{A}$ and/or ${}^{B}{\text{V}}_{A}^{⊤}$. Note the analogy between (19) and the hybrid position/force control framework (7).

We will use this classification to characterize the works reviewed in the rest of this Section.

### 4.1. Traded Control

The paper (Cherubini et al., 2016) presented a human-robot manufacturing cell for collaborative assembly of car joints. The approach (traded *vision/touch*) could manage physical contact between robot and human, and between robot and environment, via admittance control (8). Vision would take over in dangerous situations to trigger emergency stops. The switching *condition* was determined by the position of the human wrt the robot.

In Okuno et al. (2001, 2004), a traded *vision/audio* controller enabled a mobile robot to exploit sound source localization for visual control. The robot head would automatically rotate toward the estimated direction of the human speaker, and then visually track him/her. The switching *condition* is that the sound source is visible. The audio-based task is equivalent to regulating τ to 0 or ρ to 1, as discussed in section 3.4. Paper (Hornstein et al., 2006) presented another traded *vision/audio* controller for the iCub robot head to localize a human speaker. This method constructed audio-motor maps and integrated visual feedback to update the map. Again, the switching *condition* is that the speaker's face is visible. In Chan et al. (2012), another traded *vision/audio* controller was deployed on a mobile robot, to drive it toward an unknown sound source; the switching *condition* is defined by a threshold on the frontal localization error.

The authors of Papageorgiou et al. (2014) presented a mobile assistant for people with walking impairments. The robot was equipped with: two wrench sensors to measure physical interaction with the human, an array of microphones for audio commands, laser sensors for detecting obstacles, and an RGB-D camera for estimating the users' state. Its controller integrated *audio, touch, vision*, and *distance* in a traded manner, with switching *conditions* determined by a knowledge-based layer.

The work (Navarro et al., 2014) presented an object manipulation strategy, integrating *distance* (capacitive proximity sensors) and *touch* (tactile sensors). While the method did not explicitly consider humans, it may be applied for human-robot collaboration, since proximity sensors can detect humans if vision is occluded. The switching *condition* between the two modes is the contact with the object.

Another example of traded control—here, *audio/distance*—is Huang et al. (1999), which presented a method for driving a mobile robot toward hidden sound sources, via an omnidirectional array of microphones. The controller switched to ultrasound-based obstacle avoidance in the presence of humans/objects. The detection of a nearby obstacle is the switching *condition*.

### 4.2. Shared Control

In applications where the robot and environment/human are in permanent contact (e.g., collaborative object transportation), shared control is preferable. Let us first review a pioneer controller (Morel et al., 1998) that relied on shared *vision/touch*, as outlined in Figure 3; Morel et al. (1998) addressed tele-operated peg-in-hole assembly, by placing the visual loop outside the force loop. The reference trajectory ${\overset{∙}{\text{x}}}^{r}$ output by visual servoing was deformed in the presence of contact by the admittance controller, to obtain the robot position command **x**. Human interaction was not considered in this work.

The authors of Natale et al. (2002) estimated sensory-motor responses to control a pan-tilt robot head with shared *visual/audio* feedback from humans. They assumed local linear relations between the robot motions and the ITD/ILD measures. This resulted in a controller which is simpler than the one presented in section 3.4. The scheme is similar to Figure 3, with an outer *vision* loop generating a reference motion, and *audio* modifying it.

### 4.3. Hybrid Control

Pomares et al. (2011) proposed a hybrid *vision/touch* controller for grasping objects, using a robot arm equipped with a hand. Visual feedback drives an active camera (installed on the robot tip) to observe the object and detect humans to be avoided, whereas touch feedback moves the fingers, to grasp the object. The authors defined matrix **S** in (7) to independently control arm and fingers with the respective sensor.

In Chatelain et al. (2017), a hybrid scheme controlled an ultrasonic probe in contact with the abdomen of a patient. The goal was to center the lesions in the ultrasound image observed by the surgeon. The probe was moved by projecting, via **S**, the *touch*, and *vision* (from the ultrasound image) tasks in orthogonal directions.

### 4.4. Other Control Schemes

Some works do not strictly follow the classification given above. These are reviewed below.

The authors of Agravante et al. (2013, 2014) combined *vision* and *touch* to address joint human-humanoid table carrying. The table must stay flat, to prevent objects on top from falling off. Vision controlled the table inclination, whereas the forces exchanged with the human made the robot follow his/her intention. The approach is *shared*, with visual servoing in the outer loop of admittance control (Figure 3), to make all dof compliant. However, it is also *hybrid*, since some dof are controlled only with admittance. Specifically vision regulated only the table height in Agravante et al. (2013), and both table height and roll angle in Agravante et al. (2014).

The works (Cherubini and Chaumette, 2013; Cherubini et al., 2014) merged *vision* and *distance* to guarantee lidar-based obstacle avoidance during camera-based navigation. While following a pre-taught path, the robot must avoid obstacles which were not present before. Meanwhile, it moves the camera pan angle, to maintain scene visibility. Here, the selection matrix in (19) was a scalar function **S** ∈ [0, 1] dependent on the time-to-collision. In the safe context (**S** = 0), the robot followed the taught path, with camera looking forward. In the unsafe context (**S** = 1) the robot circumnavigated the obstacles. Therefore, the scheme is *hybrid* when **S** = 0 or **S** = 1 (i.e., vision and distance operate on independent components of the task vector), and *shared* when **S** ∈ (0, 1).

In Dean-Leon et al. (2016), proximity (*distance*) and tactile (*touch*) measurements controlled a robot arm in a pHRI scenario to avoid obstacles or—when contact is inevitable—to generate compliant behaviors. The framework linearly combined the two senses, and provided this signal to an inner admittance-like control loop (as in the *shared* scheme of Figure 3). Since the operation principle of both senses was complementary (one requires contact while the other does not), the integration can also be seen as *traded*.

The authors of Cherubini et al. (2015) enabled a robot to adapt to changes in the human behavior, during a human-robot collaborative screwing task. In contrast with classic hybrid vision–touch–position control, their scheme enabled smooth transitions, via weighted combinations of the tasks. The robot could execute *vision* and *force* tasks, either exclusively on different dof (*hybrid* approach) or simultaneously (*shared* approach).

## 5. Classification of Works and Discussion

In this section, we use five criteria to classify all the surveyed papers which apply sensor-based control to collaborative robots. This taxonomy then serves as an inspiration to drive the following discussion on design choices, limitations, and future challenges.

In total, we refer to the 45 papers revised above. These include the works with only one sensor, discussed in section 3 (Maeda et al., 2001; Kumon et al., 2003, 2005; De Santis et al., 2007; Dune et al., 2008; Suphi Erden and Tomiyama, 2010; Suphi Erden and Maric, 2011; Tsui et al., 2011; Bussy et al., 2012; Flacco et al., 2012; Youssef et al., 2012; Ficuciello et al., 2013; Schlegl et al., 2013; Agustinos et al., 2014; Baumeyer et al., 2015; Gridseth et al., 2015, 2016; Magassouba et al., 2015, 2016a,b,c; Wang et al., 2015; Bauzano et al., 2016; Cai et al., 2016; Leboutet et al., 2016; Narayanan et al., 2016; Bergner et al., 2017; Cortesao and Dominici, 2017; Dean-Leon et al., 2017) and those which integrated multiple sensors, discussed in section 4 (Huang et al., 1999; Okuno et al., 2001, 2004; Natale et al., 2002; Hornstein et al., 2006; Pomares et al., 2011; Chan et al., 2012; Cherubini and Chaumette, 2013; Cherubini et al., 2014, 2015, 2016; Navarro et al., 2014; Papageorgiou et al., 2014; Dean-Leon et al., 2016; Chatelain et al., 2017). The five criteria are: sensor(s), integration method (when multiple sensors are used), control objective, target sector, and robot platform. In Table 1, we indicate these characteristics for each paper. Then, we focus on each characteristic, in Tables 2–**5**^4^.

**Table 1:** Classification of all papers according to four criteria: sense(s) used by the robot, objective of the controller, target sector, and type of robot.

**References**	**Sense(s)**	**Control objective**	**Sector**	**Robot**
Cai et al. (2016) and Gridseth et al. (2016)	Vision	Contactless guidance	Service	Arm
Gridseth et al. (2015)	Vision	Remote guidance	Service	Arm
Dune et al. (2008), Tsui et al. (2011), and Narayanan et al. (2016)	Vision	Contactless guidance	Medical	Wheeled
Agustinos et al. (2014)	Vision	Contact w/humans	Medical	Arm
Bauzano et al. (2016)	Touch	Contact w/humans	Medical	Arm
		Remote guidance		
Cortesao and Dominici (2017)	Touch	Contact w/humans	Medical	Arm
Maeda et al. (2001), Suphi Erden and Tomiyama (2010), Suphi Erden and Maric (2011), and Ficuciello et al. (2013)	Touch	Direct guidance	Production	Arm
Wang et al. (2015)	Touch	Carrying	Production	Wheeled
Bussy et al. (2012)	Touch	Carrying	Production	Humanoid
Baumeyer et al. (2015)	Touch	Remote guidance	Medical	Arm
Kumon et al. (2003, 2005), Magassouba et al. (2016b)	Audition	Contactless guidance	Service	Heads
Magassouba et al. (2015, 2016a,c)	Audition	Contactless guidance	Service	Wheeled
De Santis et al. (2007), Flacco et al. (2012), and Schlegl et al. (2013)	Distance	Collision avoidance	Production	Arm
Leboutet et al. (2016), Bergner et al. (2017), and Dean-Leon et al. (2017)	Distance	Collision avoidance	Service	Arm
Cherubini et al. (2016)	V+T (tra.)	Assembly	Production	Arm
Okuno et al. (2001), Okuno et al. (2004), and Hornstein et al. (2006)	V+A(tra.)	Contactless guidance	Service	Heads
Chan et al. (2012)	V+A(tra.)	Contactless guidance	Service	Wheeled
Papageorgiou et al. (2014)	V+T+A+D	Direct guidance	Medical	Wheeled
	(tra.)			
Navarro et al. (2014)	D+T(tra.)	Collision avoidance	Production	Arm
Huang et al. (1999)	D+A(tra.)	Collision avoidance	Service	Wheeled
Natale et al. (2002)	V+A(sh.)	Contactless guidance	Service	Heads
Pomares et al. (2011)	V+T(hyb.)	Collision avoidance	Production	Arm
Chatelain et al. (2017)	V+T	Contact w/humans	Medical	Arm
	(hyb.)	Remote guidance		
Agravante et al. (2013, 2014)	V+T	Contact w/humans	Production	Humanoid
	(sh.+hyb.)			
Cherubini and Chaumette (2013), Cherubini et al. (2014)	D+V	Collision avoidance	Production	Wheeled
	(sh.+hyb.)			
Dean-Leon et al. (2016)	D+T	Direct guidance	Service	Arm
	(sh.+tra.)			
Cherubini et al. (2015)	V+T	Assembly	Production	Arm
	(sh.+hyb.)			

**Table 2:** Classification based on the sensors.

Vision	Dune et al., 2008; Tsui et al., 2011; Agustinos et al., 2014; Gridseth et al., 2015, 2016; Cai et al., 2016; Narayanan et al., 2016			
Touch	Maeda et al., 2001; Suphi Erden and Tomiyama, 2010; Suphi Erden and Maric, 2011; Bussy et al., 2012; Ficuciello et al., 2013; Baumeyer et al., 2015; Wang et al., 2015; Bauzano et al., 2016; Cortesao and Dominici, 2017	tra. (Cherubini et al., 2016), hyb. (Pomares et al., 2011; Chatelain et al., 2017) sh.+hyb. (Agravante et al., 2013, 2014; Cherubini et al., 2015)		
Audition	Kumon et al. (2003, 2005); Youssef et al. (2012); Magassouba et al. (2015, 2016a,b,c)	tra. Okuno et al. (2001, 2004); Hornstein et al. (2006); Chan et al. (2012); Papageorgiou et al. (2014), sh. (Natale et al., 2002)	tra. (Papageorgiou et al., 2014)	
Distance	De Santis et al., 2007; Flacco et al., 2012; Schlegl et al., 2013; Leboutet et al., 2016; Bergner et al., 2017; Dean-Leon et al., 2017	sh.+hyb. (Cherubini and Chaumette, 2013; Cherubini et al., 2014)	sh.+tra. (Dean-Leon et al., 2016)	tra. (Huang et al., 1999; Papageorgiou et al., 2014)
			tra. (Navarro et al., 2014)	
	Mono	Vision	Touch	Audition

Table 2 classifies the papers according to the sensor/s. Column *mono* indicates the papers relying only on one sensor. For the others, we specify the integration approach (see section 4). Note that vision (alone or not) is by far the most popular sense, used in 22 papers. This comes as no surprise, since even for humans, vision provides the richest perceptual information to structure the world and perform motion (Hoffman, 1998). Touch is the second most commonly used sensor (18 papers) and fundamental in pHRI, since it is the only one among the four that can be exploited directly to modulate contact.

Also note that, apart from Papageorgiou et al. (2014), no paper integrates more than two sensors. Given the sensors wide accessibility and the recent progress in computation power, this is probably due to the difficulty in designing a framework capable of managing such diverse and broad data. Another reason may be the presumed (but disputable) redundancy of the three contact-less senses, which biases toward opting for vision, given its diffusion and popularity (also in terms of software). Touch—the only sensor measuring contact—is irreplaceable. This may also be the reason why, when merging two sensors, researchers have generally opted for vision+touch (7 out of 17 papers). The most popular among the three integration methods is *traded control*, probably because it is the easiest to set up. In recent years, however, there has been a growing interest toward the *shared+hybrid* combination, which guarantees nice properties in terms of control smoothness.

An unexploited application of shared control is the combination of *vision* and *distance* (proximity sensors) to avoid collisions with humans. This can be formulated as in Figure 3 by replacing touch control error **e_t_** with an admittance-like distance control error:

(20) $$\begin{array}{r} {\text{e}}_{d}=-\left(\text{d}-{\text{d}}^{*}\right)+\text{M}\left(\ddot{\text{x}}-{\overset{∙}{\text{x}}}^{r}\right)+\text{B}\left(\overset{∙}{\text{x}}-{\overset{∙}{\text{x}}}^{r}\right)+\text{K}\left(\text{x}-{\text{x}}^{r}\right), \end{array}$$

where **d** and d^*^ represent the measured and desired distance to obstacles. With this approach, the robot can stabilize at a given “safe” distance from an obstacle, or move away from it.

In the authors' opinion, no sensor(s) nor (if needed) integration method is the best, and the designer should choose according to the objective at stake. For this, nature and evolution can be extremely inspiring but technological constraints (e.g., hardware and software availability) must also be accounted for, with the golden rule of engineering that “simpler is better.”

Table 3 classifies the papers according to the *control objective*. In the table, we also apply the taxonomy of pHRI layers introduced in De Luca and Flacco (2012), and evoked in the introduction: *safety, coexistence, collaboration*. Works that focus on collision avoidance address *safety*, and works where the robot acts on passive humans address *coexistence*. For the *collaboration* layer, we distinguish two main classes of works. First, those where the human was guiding the robot (without contact, with direct contact, or with remote physical contact as in tele-operation), then those where the two collaborated (e.g., for part assembly or object carrying). The idea (also in line with De Luca and Flacco, 2012) is the lower lines in the table generally require higher cognitive capabilities (e.g., better modeling of environment and task). Some works, particularly in the field of medical robotics (Agustinos et al., 2014; Bauzano et al., 2016; Chatelain et al., 2017) cover both coexistence and collaboration, since the human guided the robot to operate on another human. Interestingly, the senses appear in the table with a trend analogous to biology. *Distance* is fundamental for collision avoidance, when the human is far, and his/her role in the interaction is basic (s/he is mainly perceived as an obstacle). Then, audio is used for contactless guidance. As human and robot are closer, *touch* takes over the role of *audio*. As mentioned above, *vision* is a transversal sense, capable of covering most distance ranges. Yet, when contact is present (i.e., in the four lower lines), it is systematically complemented by touch, a popular pairing as also shown in Table 2 and discussed above.

**Table 3:** Classification based on the control objective with corresponding pHRI layer as proposed in De Luca and Flacco (2012) (in parenthesis).

Collision avoidance (*safety*)	Distance (De Santis et al., 2007; Flacco et al., 2012; Schlegl et al., 2013; Leboutet et al., 2016; Bergner et al., 2017; Dean-Leon et al., 2017), distance+touch (Navarro et al., 2014),
	Distance+audition (Huang et al., 1999), vision+touch (Pomares et al., 2011),
	Vision+distance (Cherubini and Chaumette, 2013; Cherubini et al., 2014)
Contact with passive humans (*coexistence*)	Vision (Agustinos et al., 2014), touch (Bauzano et al., 2016; Cortesao and Dominici, 2017),
	Vision+touch (Chatelain et al., 2017)
Contactless guidance (*collaboration*)	Vision (Dune et al., 2008; Tsui et al., 2011; Cai et al., 2016; Gridseth et al., 2016; Narayanan et al., 2016)
	Audition (Kumon et al., 2005; Youssef et al., 2012; Magassouba et al., 2015, 2016a,b,c)
	Vision+audition (Okuno et al., 2001, 2004; Natale et al., 2002; Hornstein et al., 2006; Chan et al., 2012)
Direct guidance (*collaboration*)	Touch+audition+distance+vision (Papageorgiou et al., 2014),
	Touch (Maeda et al., 2001; Suphi Erden and Tomiyama, 2010; Suphi Erden and Maric, 2011; Ficuciello et al., 2013), touch+distance (Dean-Leon et al., 2016)
Remote guidance (*collaboration*)	Vision (Agustinos et al., 2014; Gridseth et al., 2015), touch (Baumeyer et al., 2015; Bauzano et al., 2016),
	Vision+touch (Chatelain et al., 2017)
Collaborative assembly (*collaboration*)	Vision+touch (Cherubini et al., 2015, 2016)
Collaborative carrying (*collaboration*)	Touch (Bussy et al., 2012; Wang et al., 2015),
	vision+touch (Agravante et al., 2013, 2014)

Table 4 classifies the papers according to the target (or *potential*) sector. We propose three sectors: *Production, Medical*, and *Service*. Production is the historical sector of robotics; applications include: manufacturing (assembly, welding, pick-and-place), transportation (autonomous guided vehicles, logistics) and construction (material and brick transfer). The medical category has become very popular in recent years, with applications spanning from robotic surgery (surgical grippers and needle manipulation), diagnosis (positioning of ultrasonic probes; Tirindelli et al., 2020 or endoscopes), and assistance (intelligent wheelchairs, feeding and walking aids). The service sector is the one that in the authors' opinion presents the highest potential for growth in the coming years. Applications include companionship (elderly and child care), domestic (cleaning, object retrieving), personal (chat partners, tele-presence). The table shows that all four sensors have been deployed in all three sectors. The only exception is *audition* not being used in *production* applications, probably because of the noise—common in industrial environments.

**Table 4:** Classification based on target/potential sectors.

*Production* (manufacturing, transportation, construction)	Touch (Maeda et al., 2001; Suphi Erden and Tomiyama, 2010; Suphi Erden and Maric, 2011; Bussy et al., 2012; Ficuciello et al., 2013; Wang et al., 2015), distance (De Santis et al., 2007; Flacco et al., 2012; Schlegl et al., 2013),
	D+T (Navarro et al., 2014) V+T (Pomares et al., 2011; Agravante et al., 2013, 2014; Cherubini et al., 2015, 2016),
	V+D (Cherubini and Chaumette, 2013; Cherubini et al., 2014)
*Medical* (surgery, diagnosis, assistance)	Vision (Dune et al., 2008; Tsui et al., 2011; Agustinos et al., 2014; Narayanan et al., 2016), touch (Baumeyer et al., 2015; Bauzano et al., 2016; Cortesao and Dominici, 2017),
	V+T+A+D (Papageorgiou et al., 2014), V+T (Chatelain et al., 2017)
*Service* (companionship, domestic, personal)	Vision (Gridseth et al., 2015, 2016; Cai et al., 2016), audition (Kumon et al., 2005; Youssef et al., 2012; Magassouba et al., 2015, 2016a,b,c),
	distance (Leboutet et al., 2016; Bergner et al., 2017; Dean-Leon et al., 2017), V+A (Okuno et al., 2001, 2004; Natale et al., 2002; Hornstein et al., 2006; Chan et al., 2012),
	D+A (Huang et al., 1999), T+D (Dean-Leon et al., 2016)

Finally, Table 5 gives a classification based on the robotic platform. We can see that (unsurprisingly) most works use fixed base *arms*. The second most used platforms here are *wheeled* robots. Then, the *humanoids* category, which refers to robots with anthropomorphic design (two arms and biped locomotion capabilities). Finally, we consider robot *heads*, which are used exclusively for audio-based control. While robot heads are commonly used for face tracking in *Social Human Robot Interaction*, such works are not reviewed in this survey as they do not generally involve contact.

**Table 5:** Classification based on the type of robot platform.

Arms	Vision (Agustinos et al., 2014; Gridseth et al., 2015, 2016; Cai et al., 2016), touch (Maeda et al., 2001; Suphi Erden and Tomiyama, 2010; Suphi Erden and Maric, 2011; Ficuciello et al., 2013; Baumeyer et al., 2015; Bauzano et al., 2016; Cortesao and Dominici, 2017), distance (De Santis et al., 2007; Flacco et al., 2012; Schlegl et al., 2013; Leboutet et al., 2016; Bergner et al., 2017; Dean-Leon et al., 2017),
	V+T (Pomares et al., 2011; Cherubini et al., 2015, 2016; Chatelain et al., 2017), D+T (Navarro et al., 2014; Dean-Leon et al., 2016)
Wheeled	Vision (Dune et al., 2008; Tsui et al., 2011; Narayanan et al., 2016), touch (Wang et al., 2015), audition (Magassouba et al., 2015, 2016a,b), V+A (Chan et al., 2012), V+T+A+D (Papageorgiou et al., 2014), D+A (Huang et al., 1999), V+D (Cherubini and Chaumette, 2013; Cherubini et al., 2014)
Humanoids	Touch (Bussy et al., 2012), V+T (Agravante et al., 2013, 2014)
Heads	Audition (Kumon et al., 2003, 2005; Magassouba et al., 2016b), V+A (Okuno et al., 2001, 2004; Natale et al., 2002; Hornstein et al., 2006)

## 6. Conclusions

This work presents a systematic review of sensor-based controllers which enable collaboration and/or interaction between humans and robots. We considered four senses: vision, touch, audition, and distance. First, we introduce a tutorial-like general formulation of sensor-based control (Navarro-Alarcon et al., 2020), which we instantiate for visual servoing, touch control, aural servoing, and distance-based control, while reviewing representative papers. Next, with the same formulation, we model the methods that integrate multiple sensors, while again discussing related works. Finally, we classify the surveyed body of literature according to: used sense(s), integration method, control objective, target application, and platform.

Although vision and touch (*proprioceptive force* rather than *tact*) emerge nowadays as the most popular senses on collaborative robots, the advent of cheap, precise, and easy to integrate tactile, distance, and audio sensors present great opportunities for the future. Typically, we believe that robot skins (e.g., on arms and hands, Guadarrama-Olvera et al., 2019; Navarro et al., 2020) will simplify interaction, boosting the opportunities for human-robot collaboration. It is imperative that researchers develop the appropriate tools for this. Distance/proximity feedback is promising to fully perceive the human operating near the robot (something monocular vision cannot do). Audio feedback is key for developing robotic heads that can interact in a natural way with human speakers.

Finally, some open problems must be addressed, to develop robust controllers for real-world applications. For example, the use of task constraints has not been sufficiently explored when multiple sensors are integrated. Also, difficulty in obtaining models describing and predicting human behavior hampers the implementation of human-robot collaborative tasks. The use of multimodal data such as RGB-D cameras with multiple proximity sensors may be an interesting solution for this human motion sensing and estimation problem. More research needs to be conducted in this direction.

## Data Availability Statement

The original contributions presented in the study are included in the article/supplementary materials, further inquiries can be directed to the corresponding author/s.

## Author Contributions

AC conceived the study and drafted the manuscript. DN-A revised the paper. Both authors contributed to the article and approved the submitted version.

## Conflict of Interest

The authors declare that the research was conducted in the absence of any commercial or financial relationships that could be construed as a potential conflict of interest.

## Footnotes

^1^Throughout the paper, λ is a positive tuning scalar that determines the convergence rate of task error **e** to ****0.

^2^Also known as *interaction matrix* in the visual servoing literature.

^3^Or its frequency counterpart: Interaural Phase Difference (IPD).

^4^In the Tables, we have used the following notation: V, T, A, D for Vision, Touch, Audition, and Distance, and sh., hyb., tra. for shared, hybrid, and traded.

## References

[B1] Abderrahmane Z. Ganesh G. Crosnier A. Cherubini A. (2018). Haptic zero-shot learning: recognition of objects never touched before. Robot. Auton. Syst. 105, 11–25. 10.1016/j.robot.2018.03.002

[B2] Agravante D. J. Cherubini A. Bussy A. Gergondet P. Kheddar A. (2014). “Collaborative human-humanoid carrying using vision and haptic sensing,” in IEEE Int. Conf. on Robotics and Automation, ICRA. 10.1109/ICRA.2014.6906917

[B3] Agravante D. J. Cherubini A. Bussy A. Kheddar A. (2013). “Human-humanoid joint haptic table carrying task with height stabilization using vision,” in IEEE/RSJ Int. Conf. on Robots and Intelligent Systems, IROS. 10.1109/IROS.2013.6697019

[B4] Agustinos A. Wolf R. Long J. A. Cinquin P. Voros S. (2014). “Visual servoing of a robotic endoscope holder based on surgical instrument tracking,” in IEEE RAS/EMBS Int. Conf. on Biomedical Robotics and Biomechatronics, 13–18. 10.1109/BIOROB.2014.6913744

[B5] Ajoudani A. Zanchettin A. M. Ivaldi S. Albu-Schäffer A. Kosuge K. Khatib O. (2017). Progress and prospects of the human-robot collaboration. Auton. Robots 42, 957–975. 10.1007/s10514-017-9677-2

[B6] Argall B. D. Billard A. G. (2010). A survey of tactile human-robot interactions. Robot. Auton. Syst. 58, 1159–1176. 10.1016/j.robot.2010.07.002

[B7] Azizian M. Khoshnam M. Najmaei N. Patel R. V. (2014). Visual Servoing in medical robotics: a survey. Part I: endoscopic and direct vision imaging-techniques and applications. Int. J. Med. Robot. 10, 263–274. 10.1002/rcs.1531 24106103

[B8] Baeten J. Bruyninckx H. De Schutter J. (2003). Integrated vision/force robotic servoing in the task frame formalism. Int. J. Robot. Res. 22, 941–954. 10.1177/027836490302210010

[B9] Baumeyer J. Vieyres P. Miossec S. Novales C. Poisson G. Pinault S. (2015). “Robotic co-manipulation with 6 DOF admittance control: application to patient positioning in proton-therapy,” in IEEE Int. Work. on Advanced Robotics and its Social Impacts, 1–6. 10.1109/ARSO.2015.7428220

[B10] Bauzano E. Estebanez B. Garcia-Morales I. Munoz V. F. (2016). Collaborative human-robot system for HALS suture procedures. IEEE Syst. J. 10, 957–966. 10.1109/JSYST.2014.2299559

[B11] Bergner F. Dean-Leon E. Cheng G. (2017). “Efficient event-driven reactive control for large scale robot skin,” in IEEE Int. Conf. on Robotics and Automation, ICRA, 394–400. 10.1109/ICRA.2017.7989051

[B12] Berthoz A. (2002). The Brain's Sense of Movement. Harvard University Press.

[B13] Bicchi A. Peshkin M. Colgate J. (2008). Safety for Physical Human-Robot Interaction. Springer Handbook of Robotics. 10.1007/978-3-540-30301-5_58

[B14] Bussy A. Kheddar A. Crosnier A. Keith F. (2012). “Human-humanoid haptic joint object transportation case study,” in IEEE/RSJ Int. Conf. on Robots and Intelligent Systems, IROS, 3633–3638. 10.1109/IROS.2012.6385921

[B15] Cai C. Somani N. Knoll A. (2016). Orthogonal image features for visual servoing of a 6-dof manipulator with uncalibrated stereo cameras. IEEE Trans. Robot. 32, 452–461. 10.1109/TRO.2016.2535443

[B16] Chan V. Jin C. van Schaik A. (2012). Neuromorphic audio-visual sensor fusion on a sound-localising robot. Front. Neurosci. 6:21. 10.3389/fnins.2012.00021 22347165 PMC3274764

[B17] Chatelain P. Krupa A. Navab N. (2017). Confidence-driven control of an ultrasound probe. IEEE Trans. Robot. 33, 1410–1424. 10.1109/TRO.2017.2723618

[B18] Chaumette F. Hutchinson S. (2006). Visual servo control, Part I: basic approaches. IEEE Robot. Autom. Mag. 13, 82–90. 10.1109/MRA.2006.250573

[B19] Cherubini A. Chaumette F. (2013). Visual navigation of a mobile robot with laser-based collision avoidance. Int. J. Robot. Res. 32, 189–209. 10.1177/0278364912460413

[B20] Cherubini A. Passama R. Crosnier A. Lasnier A. Fraisse P. (2016). Collaborative manufacturing with physical human-robot interaction. Robot. Comput. Integr. Manufact. 40, 1–13. 10.1016/j.rcim.2015.12.007

[B21] Cherubini A. Passama R. Fraisse P. Crosnier A. (2015). A unified multimodal control framework for human-robot interaction. Robot. Auton. Syst. 70, 106–115. 10.1016/j.robot.2015.03.002

[B22] Cherubini A. Spindler F. Chaumette F. (2014). Autonomous visual navigation and laser-based moving obstacle avoidance. IEEE Trans. Int. Transport. Syst. 15, 2101–2110. 10.1109/TITS.2014.2308977

[B23] Colgate J. Wannasuphoprasit W. Peshkin M. (1996). “Cobots: robots for collaboration with human operators,” in Proc ASME Dynamic Systems and Control Division, Vol. 58, 433–439.

[B24] Cortesao R. Dominici M. (2017). Robot force control on a beating heart. IEEE/ASME Trans. Mechatron. 22, 1736–1743. 10.1109/TMECH.2017.2696259

[B25] Davison E. Goldenberg A. (1975). Robust control of a general servomechanism problem: the servo compensator. IFAC Proc. 8, 231–239. 10.1016/S1474-6670(17)67744-9

[B26] De Luca A. Flacco F. (2012). “Integrated control for pHRI: collision avoidance, detection, reaction and collaboration,” in IEEE RAS/EMBS Int. Conf. on Biomedical Robotics and Biomechatronics, BIOROB. 10.1109/BioRob.2012.6290917

[B27] De Santis A. Lippiello V. Siciliano B. Villani L. (2007). Human-robot interaction control using force and vision. Adv. Control Theor. Appl. 353, 51–70. 10.1007/978-3-540-70701-1_3

[B28] Dean-Leon E. Bergner F. Ramirez-Amaro K. Cheng G. (2016). “From multi-modal tactile signals to a compliant control,” in IEEE-RAS Int. Conf. on Humanoid Robots, 892–898. 10.1109/HUMANOIDS.2016.7803378

[B29] Dean-Leon E. Pierce B. Bergner F. Mittendorfer P. Ramirez-Amaro K. Burger W. (2017). “TOMM: Tactile omnidirectional mobile manipulator,” in IEEE Int. Conf. on Robotics and Automation, ICRA, 2441–2447. 10.1109/ICRA.2017.7989284

[B30] Del Prete A. Denei S. Natale L. Mastrogiovanni F. Nori F. Cannata G. (2011). “Skin spatial calibration using force/torque measurements,” in IEEE/RSJ Int. Conf. on Robots and Intelligent Systems. 10.1109/IROS.2011.6094896

[B31] Dune C. Remazeilles A. Marchand E. Leroux C. (2008). “Vision-based grasping of unknown objects to improve disabled people autonomy,” in Robotics: Science and Systems.

[B32] Ernst M. O. Banks M. S. (2002). Humans integrate visual and haptic information in a statistically optimal fashion. Nature 415, 429–433. 10.1038/415429a 11807554

[B33] Ficuciello F. Romano A. Villani L. Siciliano B. (2013). “Cartesian impedance control of redundant manipulators for human-robot co-manipulation,” in IEEE/RSJ Int. Conf. on Robots and Intelligent Systems, IROS. 10.1109/IROS.2014.6942847

[B34] Flacco F. Kroger T. De Luca A. Khatib O. (2012). “A depth space approach to human-robot collision avoidance,” in IEEE Int. Conf. on Robotics and Automation, ICRA. 10.1109/ICRA.2012.6225245

[B35] Gao B. Li H. Li W. Sun F. (2016). 3D moth-inspired chemical plume tracking and adaptive step control strategy. Adapt. Behav. 24, 52–65. 10.1177/1059712315623998

[B36] Göger D. Blankertz M. Wörn H. (2010). A tactile proximity sensor. IEEE Sensors 589–594.

[B37] Gridseth M. Hertkorn K. Jagersand M. (2015). “On visual servoing to improve performance of robotic grasping,” in Conf. on Computer and Robot Vision, 245–252. 10.1109/CRV.2015.39

[B38] Gridseth M. Ramirez O. Quintero C. P. Jagersand M. (2016). “Vita: Visual task specification interface for manipulation with uncalibrated visual servoing,” in IEEE Int. Conf. on Robotics and Automation, ICRA. 10.1109/ICRA.2016.7487521

[B39] Guadarrama-Olvera J. R. Dean-Leon E. Bergner F. Cheng G. (2019). Pressure-driven body compliance using robot skin. IEEE Robot. Autom. Lett. 4, 4418–4423. 10.1109/LRA.2019.2928214

[B40] Ha D. Sun Q. Su K. Wan H. Li H. Xu N. (2015). Recent achievements in electronic tongue and bioelectronic tongue as taste sensors. Sensors Actuat. Part B 207, 1136–1146. 10.1016/j.snb.2014.09.077

[B41] Haddadin S. De Luca A. Albu-Schäffer A. (2017). Robot collisions: a survey on detection, isolation, and identification. IEEE Trans. Robot. 33, 1292–1312. 10.1109/TRO.2017.2723903

[B42] Hoffman D. (1998). Visual Intelligence: How We Create what We See. W. W. Norton and Company.

[B43] Hogan N. (1985). Impedance control: an approach to manipulation: parts I-III. ASME J. Dyn. Syst. Measure. Control 107, 1–24. 10.1115/1.3140701

[B44] Hornstein J. Lopes M. Santos-Victor J. Lacerda F. (2006). “Sound localization for humanoid robots - building audio-motor maps based on the HRTF,” in IEEE/RSJ Int. Conf. on Robots and Intelligent Systems, IROS, 1170–1176. 10.1109/IROS.2006.281849

[B45] Huang J. Supaongprapa T. Terakura I. Wang F. Ohnishi N. Sugie N. (1999). A model-based sound localization system and its application to robot navigation. Robot. Auton. Syst. 27, 199–209. 10.1016/S0921-8890(99)00002-0

[B46] ISO 13482:2014 (2014). Robots and Robotic Devices - Safety Requirements for Personal Care Robots. Technical report, International Organization for Standardization, Geneva.

[B47] Khatib O. (1985). “Real-time obstacle avoidance for manipulators and mobile robots,” in IEEE Int. Conf. on Robotics and Automation, ICRA. 10.1109/ROBOT.1985.1087247

[B48] Kobayashi Y. Habara M. Ikezazki H. Chen R. Naito Y. Toko K. (2010). Advanced taste sensors based on artificial lipids with global selectivity to basic taste qualities and high correlation to sensory scores. Sensors 10, 3411–3443. 10.3390/s100403411 22319306 PMC3274227

[B49] Kowadlo G. Russell R. A. (2008). Robot odor localization: a taxonomy and survey. Int. J. Robot. Res. 27, 869–894. 10.1177/0278364908095118

[B50] Kumon M. Shimoda T. Kohzawa R. Mizumoto I. Iwai Z. (2005). “Audio servo for robotic systems with pinnae,” in IEEE/RSJ Int. Conf. on Robots and Intelligent Systems, IROS, 1881–1886. 10.1109/IROS.2005.1545092

[B51] Kumon M. Sugawara T. Miike K. Mizumoto I. Iwai Z. (2003). “Adaptive audio servo for multirate robot systems,” in IEEE/RSJ Int. Conf. on Robots and Intelligent Systems, IROS, Vol. 1, 182–187. 10.1109/IROS.2003.1250625

[B52] La Valle S. M. (2006). Planning Algorithms. Cambridge University Press.

[B53] Leboutet Q. Dean-León E. Cheng G. (2016). “Tactile-based compliance with hierarchical force propagation for omnidirectional mobile manipulators,” in IEEE-RAS Int. Conf. on Humanoid Robots. 10.1109/HUMANOIDS.2016.7803383

[B54] Li Q. Schürman C. Haschke R. Ritter H. (2013). “A control framework for tactile servoing,” in Robotics: Science and Systems (RSS). 10.15607/RSS.2013.IX.045

[B55] Lin C. H. Fishel J. A. Loeb G. E. (2013). Estimating Point of Contact, Force and Torque in a Biomimetic Tactile Sensor With Deformable Skin. Technical report, SynTouch LLC.

[B56] Maeda Y. Hara T. Arai T. (2001). “Human-robot cooperative manipulation with motion estimation,” in IEEE/RSJ Int. Conf. on Robots and Intelligent Systems, IROS, Vol. 4, 2240–2245. 10.1109/IROS.2001.976403

[B57] Magassouba A. Bertin N. Chaumette F. (2015). “Sound-based control with two microphones,” in IEEE/RSJ Int. Conf. on Robots and Intelligent Systems, IROS, 5568–5573. 10.1109/IROS.2015.7354166

[B58] Magassouba A. Bertin N. Chaumette F. (2016a). “Audio-based robot control from interchannel level difference and absolute sound energy,” in IEEE/RSJ Int. Conf. on Robots and Intelligent Systems, IROS, 1992–1999. 10.1109/IROS.2016.7759314

[B59] Magassouba A. Bertin N. Chaumette F. (2016b). “Binaural auditory interaction without HRTF for humanoid robots: a sensor-based control approach,” in See, Touch, and Hear: 2nd Workshop on Multimodal Sensor-based Robot Control for HRI and Soft Manipulation, IROS.

[B60] Magassouba A. Bertin N. Chaumette F. (2016c). “First applications of sound-based control on a mobile robot equipped with two microphones,” in IEEE Int. Conf. on Robotics and Automation, ICRA, 2557–2562. 10.1109/ICRA.2016.7487411

[B61] Morel G. Malis E. Boudet S. (1998). “Impedance based combination of visual and force control,” in IEEE Int. Conf. on Robotics and Automation, ICRA, Vol. 2, 1743–1748. 10.1109/ROBOT.1998.677418

[B62] Nakadai K. Nakajima H. Murase M. Kaijiri S. Yamada K. Nakamura T. (2006). “Robust tracking of multiple sound sources by spatial integration of room and robot microphone arrays,” in IEEE Int. Conf. on Acoustics Speech and Signal Processing. 10.1109/ICASSP.2006.1661122

[B63] Narayanan K. V. Pasteau F. Marchal M. Krupa A. Babel M. (2016). Vision-based adaptive assistance and haptic guidance for safe wheelchair corridor following. Comput. Vis. Image Underst. 149, 171–185. 10.1016/j.cviu.2016.02.008

[B64] Natale L. Metta G. Sandini G. (2002). Development of auditory-evoked reflexes: visuo-acoustic cues integration in a binocular head. Robot. Auton. Syst. 39, 87–106. 10.1016/S0921-8890(02)00174-4

[B65] Natale L. Torres-Jara E. (2006). “A sensitive approach to grasping,” in Proc. of the 6th Int. Workshop on Epigenetic Robotics.

[B66] Navarro S. E. Nagels S. Alagi H. Faller L. Goury O. Morales-Bieze T. (2020). A model-based sensor fusion approach for force and shape estimation in soft robotics. IEEE Robot. Autom. Lett. 5, 5621–5628. 10.1109/LRA.2020.3008120

[B67] Navarro S. E. Schonert M. Hein B. WWörn H. (2014). “6D proximity servoing for preshaping and haptic exploration using capacitive tactile proximity sensors,” in IEEE/RSJ Int. Conf. on Robots and Intelligent Systems, IROS.

[B68] Navarro-Alarcon D. Qi J. Zhu J. Cherubini A. (2020). A Lyapunov-stable adaptive method to approximate sensorimotor models for sensor-based control. Front. Neurorobot. 14:59. 10.3389/fnbot.2020.00059 33041777 PMC7527605

[B69] Nelson B. J. Khosla P. K. (1996). Force and vision resolvability for assimilating disparate sensory feedback. IEEE Trans. Robot. Autom. 12, 714–731. 10.1109/70.538976

[B70] Nelson B. J. Morrow J. D. Khosla P. K. (1995). “Improved force control through visual servoing,” in Proc. of the American Control Conference, Vol. 1, 380–386. 10.1109/ACC.1995.529274

[B71] Nocedal J. Wright S. (2000). Numerical Optimization. Springer Series in Operations Research and Financial Engineering. 10.1007/b98874

[B72] Okuno H. G. Nakadai K. Hidai K. I. Mizoguchi H. Kitano H. (2001). “Human-robot interaction through real-time auditory and visual multiple-talker tracking,” in IEEE/RSJ Int. Conf. on Robots and Intelligent Systems, IROS, Vol. 3, 1402–1409. 10.1109/IROS.2001.977177

[B73] Okuno H. G. Nakadai K. Lourens T. Kitano H. (2004). Sound and visual tracking for humanoid robot. Appl. Intell. 20, 253–266. 10.1023/B:APIN.0000021417.62541.e0

[B74] Papageorgiou X. S. Tzafestas C. S. Maragos P. Pavlakos G. Chalvatzaki G. Moustris G. (2014). “Advances in intelligent mobility assistance robot integrating multimodal sensory processing,” in Universal Access in Human-Computer Interaction. Aging and Assistive Environments (Springer International Publishing), 692–703. 10.1007/978-3-319-07446-7_66

[B75] Passenberg C. Peer A. Buss M. (2010). A survey of environment- operator- and task-adapted controllers for teleoperation systems. Mechatronics 20, 787–801. 10.1016/j.mechatronics.2010.04.005

[B76] Phoha S. (2014). Machine perception and learning grand challenge: situational intelligence using cross-sensory fusion. Front. Robot. AI 1:7 10.3389/frobt.2014.00007

[B77] Pomares J. Perea I. Garcia G. J. Jara C. A. Corrales J. A. Torres F. (2011). A multi-sensorial hybrid control for robotic manipulation in human-robot workspaces. Sensors 11, 9839–9862. 10.3390/s111009839 22163729 PMC3231276

[B78] Proske U. Gandevia S. C. (2012). The proprioceptive senses: their roles in signaling body shape, body position and movement, and muscle force. Physiol. Rev. 92, 1651–1697. 10.1152/physrev.00048.2011 23073629

[B79] Rahbar F. Marjovi A. Kibleur P. Martinoli A. (2017). “A 3-D bio-inspired odor source localization and its validation in realistic environmental conditions,” in IEEE/RSJ Int. Conf. on Robots and Intelligent Systems, IROS, 3983–3989. 10.1109/IROS.2017.8206252

[B80] Raibert M. H. Craig J. J. (1981). Hybrid position/force control of manipulators. ASME J. Dyn. Syst. Meas. Control 126–133. 10.1115/1.3139652

[B81] Rayleigh L. (1907). On our perception of sound direction. Lond. Edinburgh Dublin Philos. Mag. J. Sci. 13, 214–232. 10.1080/14786440709463595

[B82] Russell R. A. (2006). “Tracking chemical plumes in 3-dimensions,” in IEEE Int. Conf. on Robotics and Biomimetics, 31–36. 10.1109/ROBIO.2006.340274

[B83] Schlegl T. Kröger T. Gaschler A. Khatib O. Zangl H. (2013). “Virtual whiskers-highly responsive robot collision avoidance,” in IEEE/RSJ Int. Conf. on Robots and Intelligent Systems, IROS. 10.1109/IROS.2013.6697134

[B84] Schmitz A. Maiolino P. Maggiali M. Natale L. Cannata G. Metta G. (2011). Methods and technologies for the implementation of large-scale robot tactile sensors. IEEE Trans. Robot. 27, 389–400. 10.1109/TRO.2011.2132930

[B85] Shimazu H. Kobayashi K. Hashimoto A. Kameoka T. (2007). “Tasting robot with an optical tongue: real time examining and advice giving on food and drink,” in Human Interface and the Management of Information. Methods, Techniques and Tools in Information Design, eds M. J. Smith and G. Salvendy (Berlin; Heidelberg: Springer). 10.1007/978-3-540-73345-4_107

[B86] Smits R. De Laet T. Claes K. Bruyninckx H. De Schutter J. (2008). “iTASC: a tool for multi-sensor integration in robot manipulation,” in IEEE International Conference on Multisensor Fusion and Integration for Intelligent Systems, 426–433. 10.1109/MFI.2008.4648032

[B87] Suphi Erden M. Maric B. (2011). Assisting manual welding with robot. Robot. Comput. Integr. Manufact. 27, 818–828. 10.1016/j.rcim.2011.01.00326452294

[B88] Suphi Erden M. Tomiyama T. (2010). Human intent detection and physically interactive control of a robot without force sensors. IEEE Trans. Robot. 26, 370–382. 10.1109/TRO.2010.2040202

[B89] Taylor G. Kleeman L. (2006). Visual Perception and Robotic Manipulation: 3D Object Recognition, Tracking and Hand-Eye Coordination. Springer Tracts in Advanced Robotics. Springer. 10.1007/978-3-540-33455-2

[B90] Tirindelli M. Victorova M. Esteban J. Kim S. T. Navarro-Alarcon D. Zheng Y. P. (2020). Force-ultrasound fusion: Bringing spine robotic-us to the next “level”. IEEE Robot. Autom. Lett. 5, 5661–5668. 10.1109/LRA.2020.3009069

[B91] Tsui K. M. Kim D.-J. Behal A. Kontak D. Yanco H. A. (2011). I want that: Human-in-the-loop control of a wheelchair-mounted robotic arm. Appl. Bionics Biomech. 8, 127–147. 10.1155/2011/698079

[B92] Villani L. De Schutter J. (2008). “Chapter 7: Force control,” in Springer Handbook of Robotics, eds B. Siciliano and O. Khatib (Springer), 161–185. 10.1007/978-3-540-30301-5_8

[B93] Villani V. Pini F. Leali F. Secchi C. (2018). Survey on human-robot collaboration in industrial settings: safety, intuitive interfaces and applications. Mechatronics 55, 248–266. 10.1016/j.mechatronics.2018.02.009

[B94] Wang Y. Smith C. Karayiannidis Y. Ögren P. (2015). “Cooperative control of a serial-to-parallel structure using a virtual kinematic chain in a mobile dual-arm manipulation application,” in IEEE/RSJ Int. Conf. on Robots and Intelligent Systems, IROS, 2372–2379. 10.1109/IROS.2015.7353698

[B95] Wettels N. Santos V. J. Johansson R. S. Loeb G. (2008). Biomimetic tactile sensor array. Adv. Robot. 22, 829–849. 10.1163/156855308X314533

[B96] Whitney D. (1969). Resolved motion rate control of manipulators and human prostheses. IEEE Trans. Man-Mach. Syst. 10, 47–53. 10.1109/TMMS.1969.299896

[B97] Youssef K. Argentieri S. Zarader J. L. (2012). “Towards a systematic study of binaural cues,” in IEEE/RSJ Int. Conf. on Robots and Intelligent Systems, IROS, 1004–1009. 10.1109/IROS.2012.6385554

[B98] Zhang H. Chen N. N. (2000). Control of contact via tactile sensing. IEEE Trans. Robot. Autom. 16, 482–495. 10.1109/70.880799

